# 1-Butyl-3-(1-naphtho­yl)-1*H*-indole

**DOI:** 10.1107/S1600536811016631

**Published:** 2011-05-07

**Authors:** Hong Xu, Hong-shun Sun, Feng-mao Luo, Jian-qing Ding

**Affiliations:** a Department of Chemical Engineering, Nanjing College of Chemical Technology, Geguan Road No. 265 Nanjing, Nanjing 210048, People’s Republic of China; b Department of Applied Chemistry, Nanjing College of Chemical Technology, Geguan Road No. 265 Nanjing, Nanjing 210048, People’s Republic of China; c Nanjing Xiansheng Dongyuan Pharmaceutic Company Limited, Xinglong Road No. 8 Nanjing, Nanjing 211800, People’s Republic of China; d Nanjing Sanhome Pharmaceutical Company Limited, Huizhong Road No. 9 Nanjing, Nanjing 210038, People’s Republic of China

## Abstract

In the title mol­ecule, C_23_H_21_NO, the dihedral angle between the planes of the indole ring and naphthalene ring system is 68.8 (5)°.

## Related literature

For background to cannabinoids and the synthesis of the title compound, see: Lindigkeit *et al.* (2009 ▶). For related structures, see: Bodwell *et al.* (1999 ▶).
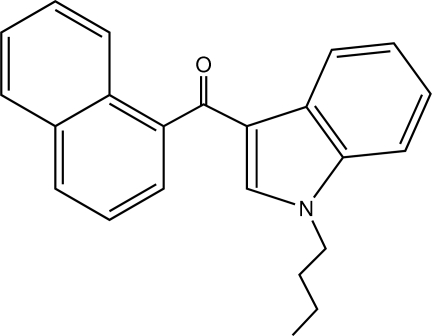


## Experimental

### 

#### Crystal data

C_23_H_21_NO
*M* *_r_* = 327.41
Orthorhombic, 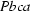

*a* = 11.799 (2) Å
*b* = 11.529 (2) Å
*c* = 26.220 (5) Å
*V* = 3566.7 (12) Å^3^
*Z* = 8
Mo *K*α radiation
μ = 0.07 mm^−1^
*T* = 293 K
0.30 × 0.20 × 0.10 mm

#### Data collection

Enraf–Nonius CAD-4 diffractometer
Absorption correction: ψ scan (North *et al.*, 1968 ▶) *T*_min_ = 0.978, *T*_max_ = 0.993
6455 measured reflections
3278 independent reflections
1527 reflections with *I* > 2σ(*I*)
*R*_int_ = 0.060
3 standard reflections every 200 reflections  intensity decay: 1%

#### Refinement

*R*[*F* ^2^ > 2σ(*F* ^2^)] = 0.060
*wR*(*F* ^2^) = 0.172
*S* = 1.00
3278 reflections
226 parameters
2 restraints
H-atom parameters constrained
Δρ_max_ = 0.23 e Å^−3^
Δρ_min_ = −0.22 e Å^−3^

### 

Data collection: *CAD-4 EXPRESS* (Enraf–Nonius, 1994 ▶); cell refinement: *CAD-4 EXPRESS*; data reduction: *XCAD4* (Harms & Wocadlo,1995) ▶; program(s) used to solve structure: *SHELXS97* (Sheldrick, 2008 ▶); program(s) used to refine structure: *SHELXL97* (Sheldrick, 2008 ▶); molecular graphics: *SHELXTL* (Sheldrick, 2008 ▶); software used to prepare material for publication: *SHELXL97*.

## Supplementary Material

Crystal structure: contains datablocks I, global. DOI: 10.1107/S1600536811016631/pv2412sup1.cif

Structure factors: contains datablocks I. DOI: 10.1107/S1600536811016631/pv2412Isup2.hkl

Supplementary material file. DOI: 10.1107/S1600536811016631/pv2412Isup3.cml

Additional supplementary materials:  crystallographic information; 3D view; checkCIF report

## Figures and Tables

**Fig. 1.**
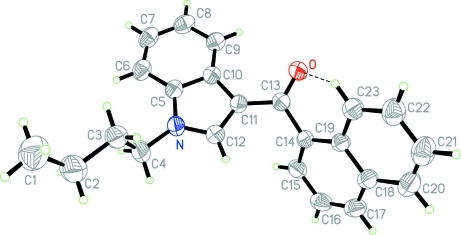
The molecular structure of the title compound, showing the atom-numbering scheme and displacement ellipsoids at the 50% probability level.

## supplementary crystallographic information

### Comment

The title compound is an analogue of JWH-018 (1-pentyl-3-(1-naphthoyl)indole), which has been banned in several European countries (Lindigkeit *et al.*, 2009). The title molecule (Fig. 1) consists of a naphthalene ring (C14—C23), an ethyl carbonyl function (O/C13) and a butylgroup (C1—C4) attached to an indole ring (N/C5—C12). The indole ring makes a dihedral angle of 68.8 (5)° with the naphthalene ring. Bond lengths and angles in the title molecule agree very well with the corresponding bond lengths and angles reported in the crystal structure of a similar compound (Bodwell *et al.*, 1999). There is an intramolecular C—H···O hydrogen bond (Fig. 1) in the molecule.

### Experimental

The title compound was synthesized with standard chemical procedures. Crystals of the title compound suitable for X-ray analysis were obtained by slow evaporation of an ethanol solution.

### Refinement

All H atoms were positioned geometrically and refined using a riding model with C—H = 0.93–0.97 Å and *U*_iso_(H) = 1.2 or 1.5 *U*_eq_(C). A rotating-group model was applied for the methyl groups.

### Crystal data

**Table:**

C_23_H_21_NO	*F*(000) = 1392
*M**_r_* = 327.41	*D*_x_ = 1.219 Mg m^−^^3^
Orthorhombic, *P**b**c**a*	Mo *K*α radiation, λ = 0.71073 Å
Hall symbol: -P 2ac 2ab	Cell parameters from 25 reflections
*a* = 11.799 (2) Å	θ = 9–13°
*b* = 11.529 (2) Å	µ = 0.07 mm^−^^1^
*c* = 26.220 (5) Å	*T* = 293 K
*V* = 3566.7 (12) Å^3^	Block, colorless
*Z* = 8	0.30 × 0.20 × 0.10 mm

### Data collection

**Table:**

Enraf–Nonius CAD-4 diffractometer	1527 reflections with *I* > 2σ(*I*)
Radiation source: fine-focus sealed tube	*R*_int_ = 0.060
graphite	θ_max_ = 25.4°, θ_min_ = 1.6°
ω/2θ scans	*h* = 0→14
Absorption correction: ψ scan (North *et al.*, 1968)	*k* = 0→13
*T*_min_ = 0.978, *T*_max_ = 0.993	*l* = −31→31
6455 measured reflections	3 standard reflections every 200 reflections
3278 independent reflections	intensity decay: 1%

### Refinement

**Table:**

Refinement on *F*^2^	Primary atom site location: structure-invariant direct methods
Least-squares matrix: full	Secondary atom site location: difference Fourier map
*R*[*F*^2^ > 2σ(*F*^2^)] = 0.060	Hydrogen site location: inferred from neighbouring sites
*wR*(*F*^2^) = 0.172	H-atom parameters constrained
*S* = 1.00	*w* = 1/[σ^2^(*F*_o_^2^) + (0.076*P*)^2^] where *P* = (*F*_o_^2^ + 2*F*_c_^2^)/3
3278 reflections	(Δ/σ)_max_ < 0.001
226 parameters	Δρ_max_ = 0.23 e Å^−^^3^
2 restraints	Δρ_min_ = −0.22 e Å^−^^3^

### Special details

**Table:**

Geometry. All e.s.d.'s (except the e.s.d. in the dihedral angle between two l.s. planes) are estimated using the full covariance matrix. The cell e.s.d.'s are taken into account individually in the estimation of e.s.d.'s in distances, angles and torsion angles; correlations between e.s.d.'s in cell parameters are only used when they are defined by crystal symmetry. An approximate (isotropic) treatment of cell e.s.d.'s is used for estimating e.s.d.'s involving l.s. planes.
Refinement. Refinement of *F*^2^ against ALL reflections. The weighted *R*-factor *wR* and goodness of fit *S* are based on *F*^2^, conventional *R*-factors *R* are based on *F*, with *F* set to zero for negative *F*^2^. The threshold expression of *F*^2^ > σ(*F*^2^) is used only for calculating *R*-factors(gt) *etc*. and is not relevant to the choice of reflections for refinement. *R*-factors based on *F*^2^ are statistically about twice as large as those based on *F*, and *R*- factors based on ALL data will be even larger.

### Fractional atomic coordinates and isotropic or equivalent isotropic displacement parameters (Å^2^)

**Table:**

	*x*	*y*	*z*	*U*_iso_*/*U*_eq_	
O	0.33878 (19)	0.1821 (2)	0.38062 (8)	0.0896 (8)	
N	0.5094 (2)	0.1691 (2)	0.22477 (10)	0.0720 (7)	
C1	0.5288 (4)	−0.0609 (5)	0.0666 (2)	0.156 (2)	
H1A	0.5807	−0.0792	0.0396	0.234*	
H1B	0.4670	−0.0160	0.0532	0.234*	
H1C	0.5000	−0.1313	0.0812	0.234*	
C2	0.5860 (4)	0.0044 (4)	0.10484 (18)	0.1259 (15)	
H2A	0.6166	0.0741	0.0894	0.151*	
H2B	0.6494	−0.0410	0.1174	0.151*	
C3	0.5113 (3)	0.0397 (3)	0.15065 (14)	0.0906 (11)	
H3A	0.4398	0.0710	0.1387	0.109*	
H3B	0.4956	−0.0275	0.1717	0.109*	
C4	0.5736 (3)	0.1292 (3)	0.18091 (13)	0.0867 (11)	
H4A	0.6449	0.0968	0.1925	0.104*	
H4B	0.5905	0.1948	0.1591	0.104*	
C5	0.4183 (3)	0.2454 (3)	0.22338 (12)	0.0654 (8)	
C6	0.3752 (3)	0.3079 (3)	0.18239 (14)	0.0816 (10)	
H6A	0.4070	0.3018	0.1500	0.098*	
C7	0.2849 (3)	0.3784 (3)	0.19135 (15)	0.0888 (11)	
H7A	0.2541	0.4207	0.1645	0.107*	
C8	0.2378 (3)	0.3884 (3)	0.23963 (15)	0.0871 (11)	
H8A	0.1759	0.4372	0.2445	0.105*	
C9	0.2806 (3)	0.3274 (3)	0.28066 (13)	0.0757 (9)	
H9A	0.2484	0.3349	0.3129	0.091*	
C10	0.3734 (2)	0.2544 (3)	0.27273 (12)	0.0641 (8)	
C11	0.4419 (3)	0.1806 (3)	0.30453 (11)	0.0641 (8)	
C12	0.5221 (3)	0.1329 (3)	0.27286 (13)	0.0735 (9)	
H12A	0.5782	0.0818	0.2836	0.088*	
C13	0.4269 (3)	0.1552 (3)	0.35786 (12)	0.0678 (8)	
C14	0.5213 (3)	0.0987 (3)	0.38654 (11)	0.0624 (8)	
C15	0.6251 (3)	0.1499 (3)	0.38816 (13)	0.0773 (10)	
H15A	0.6387	0.2150	0.3681	0.093*	
C16	0.7121 (3)	0.1070 (4)	0.41931 (14)	0.0879 (11)	
H16A	0.7820	0.1441	0.4204	0.106*	
C17	0.6931 (3)	0.0105 (4)	0.44790 (14)	0.0880 (11)	
H17A	0.7504	−0.0167	0.4691	0.106*	
C18	0.5889 (3)	−0.0496 (3)	0.44627 (12)	0.0719 (9)	
C19	0.5016 (2)	−0.0031 (3)	0.41518 (11)	0.0613 (8)	
C20	0.5676 (4)	−0.1507 (4)	0.47493 (13)	0.0901 (11)	
H20A	0.6234	−0.1799	0.4964	0.108*	
C21	0.4663 (4)	−0.2058 (3)	0.47138 (15)	0.0993 (12)	
H21A	0.4539	−0.2730	0.4902	0.119*	
C22	0.3820 (3)	−0.1639 (3)	0.44055 (15)	0.0896 (11)	
H22A	0.3136	−0.2036	0.4382	0.108*	
C23	0.3976 (3)	−0.0651 (3)	0.41350 (12)	0.0712 (9)	
H23A	0.3388	−0.0371	0.3933	0.085*	

### Atomic displacement parameters (Å^2^)

**Table:**

	*U*^11^	*U*^22^	*U*^33^	*U*^12^	*U*^13^	*U*^23^
O	0.0761 (15)	0.1134 (19)	0.0794 (16)	0.0247 (14)	0.0141 (12)	0.0163 (14)
N	0.0750 (18)	0.0827 (19)	0.0583 (17)	−0.0028 (16)	0.0049 (14)	0.0036 (14)
C1	0.166 (5)	0.167 (5)	0.137 (4)	0.020 (4)	−0.029 (4)	−0.037 (4)
C2	0.116 (3)	0.145 (4)	0.117 (3)	0.014 (3)	−0.004 (2)	−0.041 (3)
C3	0.087 (3)	0.100 (3)	0.085 (2)	0.010 (2)	−0.0014 (19)	−0.015 (2)
C4	0.085 (2)	0.106 (3)	0.069 (2)	−0.006 (2)	0.0142 (19)	0.003 (2)
C5	0.0692 (19)	0.0608 (19)	0.066 (2)	−0.0120 (18)	−0.0054 (17)	0.0082 (17)
C6	0.090 (2)	0.080 (2)	0.075 (2)	−0.018 (2)	−0.0100 (19)	0.018 (2)
C7	0.103 (3)	0.074 (2)	0.090 (3)	−0.009 (2)	−0.025 (2)	0.022 (2)
C8	0.087 (3)	0.066 (2)	0.108 (3)	0.007 (2)	−0.019 (2)	0.011 (2)
C9	0.078 (2)	0.066 (2)	0.083 (2)	0.0035 (19)	−0.0036 (18)	0.0010 (19)
C10	0.066 (2)	0.0596 (18)	0.067 (2)	−0.0090 (18)	−0.0052 (16)	0.0030 (17)
C11	0.0660 (18)	0.068 (2)	0.0585 (18)	−0.0007 (17)	−0.0009 (16)	0.0039 (16)
C12	0.067 (2)	0.084 (2)	0.069 (2)	0.0045 (18)	0.0007 (17)	0.0070 (19)
C13	0.066 (2)	0.070 (2)	0.067 (2)	0.0021 (18)	0.0031 (17)	0.0026 (17)
C14	0.0637 (19)	0.072 (2)	0.0515 (17)	0.0081 (18)	0.0028 (15)	−0.0037 (16)
C15	0.066 (2)	0.092 (2)	0.073 (2)	−0.004 (2)	0.0071 (18)	−0.0024 (19)
C16	0.061 (2)	0.117 (3)	0.086 (3)	−0.003 (2)	−0.001 (2)	−0.020 (3)
C17	0.073 (2)	0.117 (3)	0.073 (2)	0.023 (2)	−0.0098 (19)	−0.019 (2)
C18	0.077 (2)	0.084 (2)	0.0556 (19)	0.019 (2)	−0.0035 (17)	−0.0137 (19)
C19	0.0624 (19)	0.070 (2)	0.0515 (17)	0.0114 (18)	−0.0002 (15)	−0.0110 (16)
C20	0.114 (3)	0.088 (3)	0.068 (2)	0.030 (3)	−0.008 (2)	−0.002 (2)
C21	0.133 (4)	0.073 (3)	0.092 (3)	0.016 (3)	0.004 (3)	0.007 (2)
C22	0.105 (3)	0.076 (2)	0.088 (3)	0.004 (2)	0.005 (2)	0.007 (2)
C23	0.077 (2)	0.075 (2)	0.062 (2)	0.0064 (19)	0.0006 (16)	−0.0081 (18)

### Geometric parameters (Å, °)

**Table:**

O—C13	1.238 (3)	C9—H9A	0.9300
N—C12	1.337 (3)	C10—C11	1.439 (4)
N—C5	1.389 (4)	C11—C12	1.373 (4)
N—C4	1.452 (4)	C11—C13	1.440 (4)
C1—C2	1.425 (5)	C12—H12A	0.9300
C1—H1A	0.9600	C13—C14	1.494 (4)
C1—H1B	0.9600	C14—C15	1.361 (4)
C1—H1C	0.9600	C14—C19	1.412 (4)
C2—C3	1.545 (5)	C15—C16	1.402 (5)
C2—H2A	0.9700	C15—H15A	0.9300
C2—H2B	0.9700	C16—C17	1.360 (5)
C3—C4	1.494 (5)	C16—H16A	0.9300
C3—H3A	0.9700	C17—C18	1.412 (5)
C3—H3B	0.9700	C17—H17A	0.9300
C4—H4A	0.9700	C18—C20	1.410 (5)
C4—H4B	0.9700	C18—C19	1.418 (4)
C5—C6	1.391 (4)	C19—C23	1.420 (4)
C5—C10	1.402 (4)	C20—C21	1.356 (5)
C6—C7	1.360 (4)	C20—H20A	0.9300
C6—H6A	0.9300	C21—C22	1.370 (5)
C7—C8	1.388 (5)	C21—H21A	0.9300
C7—H7A	0.9300	C22—C23	1.355 (4)
C8—C9	1.381 (4)	C22—H22A	0.9300
C8—H8A	0.9300	C23—H23A	0.9300
C9—C10	1.397 (4)		
			
C12—N—C5	108.0 (3)	C9—C10—C5	118.6 (3)
C12—N—C4	126.1 (3)	C9—C10—C11	135.2 (3)
C5—N—C4	125.7 (3)	C5—C10—C11	106.2 (3)
C2—C1—H1A	109.5	C12—C11—C10	105.9 (3)
C2—C1—H1B	109.5	C12—C11—C13	126.2 (3)
H1A—C1—H1B	109.5	C10—C11—C13	127.8 (3)
C2—C1—H1C	109.5	N—C12—C11	111.6 (3)
H1A—C1—H1C	109.5	N—C12—H12A	124.2
H1B—C1—H1C	109.5	C11—C12—H12A	124.2
C1—C2—C3	114.6 (4)	O—C13—C11	121.4 (3)
C1—C2—H2A	108.6	O—C13—C14	119.5 (3)
C3—C2—H2A	108.6	C11—C13—C14	119.1 (3)
C1—C2—H2B	108.6	C15—C14—C19	119.5 (3)
C3—C2—H2B	108.6	C15—C14—C13	119.8 (3)
H2A—C2—H2B	107.6	C19—C14—C13	120.5 (3)
C4—C3—C2	108.3 (3)	C14—C15—C16	121.6 (3)
C4—C3—H3A	110.0	C14—C15—H15A	119.2
C2—C3—H3A	110.0	C16—C15—H15A	119.2
C4—C3—H3B	110.0	C17—C16—C15	119.3 (3)
C2—C3—H3B	110.0	C17—C16—H16A	120.4
H3A—C3—H3B	108.4	C15—C16—H16A	120.4
N—C4—C3	112.5 (3)	C16—C17—C18	121.8 (3)
N—C4—H4A	109.1	C16—C17—H17A	119.1
C3—C4—H4A	109.1	C18—C17—H17A	119.1
N—C4—H4B	109.1	C20—C18—C17	123.0 (4)
C3—C4—H4B	109.1	C20—C18—C19	119.3 (3)
H4A—C4—H4B	107.8	C17—C18—C19	117.7 (3)
N—C5—C6	129.1 (3)	C14—C19—C18	120.0 (3)
N—C5—C10	108.4 (3)	C14—C19—C23	122.9 (3)
C6—C5—C10	122.5 (3)	C18—C19—C23	117.0 (3)
C7—C6—C5	117.5 (3)	C21—C20—C18	120.5 (4)
C7—C6—H6A	121.2	C21—C20—H20A	119.7
C5—C6—H6A	121.2	C18—C20—H20A	119.7
C6—C7—C8	121.4 (4)	C20—C21—C22	121.0 (4)
C6—C7—H7A	119.3	C20—C21—H21A	119.5
C8—C7—H7A	119.3	C22—C21—H21A	119.5
C9—C8—C7	121.4 (4)	C23—C22—C21	120.4 (4)
C9—C8—H8A	119.3	C23—C22—H22A	119.8
C7—C8—H8A	119.3	C21—C22—H22A	119.8
C8—C9—C10	118.6 (3)	C22—C23—C19	121.6 (3)
C8—C9—H9A	120.7	C22—C23—H23A	119.2
C10—C9—H9A	120.7	C19—C23—H23A	119.2
			
C1—C2—C3—C4	−166.9 (4)	C10—C11—C13—O	11.9 (5)
C12—N—C4—C3	98.8 (4)	C12—C11—C13—C14	18.0 (5)
C5—N—C4—C3	−76.0 (4)	C10—C11—C13—C14	−166.2 (3)
C2—C3—C4—N	179.4 (3)	O—C13—C14—C15	−120.9 (3)
C12—N—C5—C6	177.7 (3)	C11—C13—C14—C15	57.2 (4)
C4—N—C5—C6	−6.7 (5)	O—C13—C14—C19	54.4 (4)
C12—N—C5—C10	−0.7 (3)	C11—C13—C14—C19	−127.5 (3)
C4—N—C5—C10	174.9 (3)	C19—C14—C15—C16	−2.8 (5)
N—C5—C6—C7	−179.3 (3)	C13—C14—C15—C16	172.5 (3)
C10—C5—C6—C7	−1.1 (5)	C14—C15—C16—C17	1.3 (5)
C5—C6—C7—C8	0.5 (5)	C15—C16—C17—C18	1.5 (5)
C6—C7—C8—C9	0.1 (5)	C16—C17—C18—C20	179.0 (3)
C7—C8—C9—C10	−0.1 (5)	C16—C17—C18—C19	−2.6 (5)
C8—C9—C10—C5	−0.6 (4)	C15—C14—C19—C18	1.5 (4)
C8—C9—C10—C11	178.5 (3)	C13—C14—C19—C18	−173.7 (3)
N—C5—C10—C9	179.7 (2)	C15—C14—C19—C23	−176.7 (3)
C6—C5—C10—C9	1.2 (4)	C13—C14—C19—C23	8.1 (4)
N—C5—C10—C11	0.4 (3)	C20—C18—C19—C14	179.5 (3)
C6—C5—C10—C11	−178.1 (3)	C17—C18—C19—C14	1.1 (4)
C9—C10—C11—C12	−179.1 (3)	C20—C18—C19—C23	−2.2 (4)
C5—C10—C11—C12	0.0 (3)	C17—C18—C19—C23	179.4 (3)
C9—C10—C11—C13	4.4 (6)	C17—C18—C20—C21	−179.1 (3)
C5—C10—C11—C13	−176.5 (3)	C19—C18—C20—C21	2.5 (5)
C5—N—C12—C11	0.7 (4)	C18—C20—C21—C22	−0.9 (6)
C4—N—C12—C11	−174.9 (3)	C20—C21—C22—C23	−1.1 (6)
C10—C11—C12—N	−0.5 (4)	C21—C22—C23—C19	1.3 (5)
C13—C11—C12—N	176.1 (3)	C14—C19—C23—C22	178.6 (3)
C12—C11—C13—O	−164.0 (3)	C18—C19—C23—C22	0.3 (4)

### Hydrogen-bond geometry (Å, °)

**Table:**

*D*—H···*A*	*D*—H	H···*A*	*D*···*A*	*D*—H···*A*
C23—H23A···O	0.93	2.55	3.057 (4)	115
